# Blood biomarkers for estimating energy intake in Japanese male collegiate athletes: a pilot study

**DOI:** 10.1186/s13102-023-00765-6

**Published:** 2023-11-08

**Authors:** Yuka Kurosaka, Takaaki Nagasawa, Kumiko Minato, Tomomi Hasegawa-Tanaka, Hisashi Naito, Sawako Wakui, Shuichi Machida

**Affiliations:** 1 Faculty of Health and Sports Science, Juntendo University, 1–1 Hirakagakuendai, Inzai, Chiba 270–1695 Japan; 2 Graduate School of Human Ecology, Wayo Women’s University, 2–3-1 Konodai, Ichikawa, Chiba 272–8533 Japan; 3 Faculty of Management and Information Sciences, Josai International University, 1 Gumyo, Togane, Chiba 283–8555 Japan; 4 Graduate School of Health and Sports Science, Juntendo University, 1–1 Hirakagakuendai, Inzai, Chiba 270–1695 Japan; 5 Institute of Health and Sports Science & Medicine, Juntendo University, 1–1 Hirakagakuendai, Inzai, Chiba 270-1695 Japan

**Keywords:** Dietary assessment, Metabolic rate, Sports nutrition

## Abstract

**Background:**

Athletes should be informed of their required energy intake in preparation for sports competitions. However, the environment in which dietary surveys can be conducted to determine the required energy intake for sports competitions is limited, and such survey will require a substantial amount of time and effort from athletes and dietitians. If certain biomarkers for estimating the energy intake can be identified, they may compensate for the shortcomings of these dietary surveys. We aimed to identify the blood biomarkers to estimate the energy intake/basal metabolic rate ratio of male athletes.

**Methods:**

Twenty-six male athletes from a university physical education department were included and underwent measurements of height, weight, and body composition, as well as blood sampling. The dietary assessment included a 3-day dietary recall and collection of meal photographs. The basal metabolic rate was estimated using the lean body mass, while the daily energy intake/basal metabolic rate ratio was used as an index to determine the energy intake. From the 36 selected blood biomarkers, we identified the independent biomarkers for inclusion in the multiple regression analysis by assessing for pairwise correlations and multicollinearity. A formula for estimating the energy intake/basal metabolic rate was then developed using the stepwise method. A *p*-value of < 0.05 was considered significant.

**Results:**

Overall, 18 of the 36 blood biomarkers were selected, and multiple regression analysis revealed that triiodothyronine, white blood cell count, and triglyceride level were significant factors that can be used to estimate the energy intake/basal metabolic rate, accounting for 60.4% of the variance. No systematic errors were observed in the estimated values, calculated using the estimation formula and dietary assessment results.

**Conclusions:**

A combination of free triiodothyronine level, white blood cell count, and triglyceride level can be used for estimating the energy intake/basal metabolic rate of male athletes, thus compensating for the shortcomings of dietary surveys.

## Background

Appropriate energy intake (EI) is among the factors that maintain an athlete’s physical function, optimally maximizes the training effect, and keeps them healthy [1]. However, the amount of energy actually ingested is difficult to determine. Usually, an athlete’s EI from food, drinks, and supplements is determined through a review of the weighed or measured food records (typically 3–7 days), multiple-pass 24-h recalls, or surveys using food frequency questionnaires. However, these assessment methods need to be performed by a trained sports dietitian, with substantial effort required from both the athlete and dietitian.

Furthermore, underreporting remains an issue associated with the use of these assessment methods, especially among athletes when the information is shared with coaches or managers [2]. A previous review examining the athletes’ self-reported EI using doubly labeled water (DLW) showed that the underreported EI accounted for approximately 10%–50% of the total EI [3]. Particularly, the EI of collegiate athletes may be underreported more frequently than that of the general population; hence, the nutrient intakes of athletes should be interpreted with caution [4]. Furthermore, recording one’s diet may inherently increase self-awareness of diet-related behaviors [2], such as restricting intake or excluding certain types of food during the monitoring period.

Epidemiological studies in the general population use specific biomarkers as surrogates for measuring the intake of selected nutrients or dietary components. These biomarkers are highly correlated with dietary intake, free of social desirability bias, and independent of memory and description of food consumed [5]. Therefore, the use of biomarkers to understand EI may be helpful, especially in collegiate athletes in whom underestimation is a problem [2] and frequent adjustment of EI is required [6].

However, a biochemical method for determining an athlete’s EI has not yet been established. Using biomarkers to understand EI may be a helpful strategy for screening excessive and insufficient EI and for providing nutrition education. Given that body composition has a large influence on energy expenditure in athletes [7], it is common practice to use the reported EI and the predicted basal metabolic rate (BMR) to estimate the amount of energy available for activity. Considering that energy requirement is usually calculated as basal metabolism × physical activity level, standardizing EI by BMR is a reasonable method. This study aimed to identify surrogate blood biomarkers that can be used to estimate the EI/BMR of male athletes. We hypothesized that the combination of multiple biomarkers can reliably estimate the EI/BMR.

## Methods

### Study design and participants

This observational cross-sectional study was approved by the Ethics Committee of Juntendo University (approval no.: 29–82, date: September 11, 2017) and Wayo Women’s University Ethics Committee on Biological and Epidemiological Studies Targeting Humans (approval no.: 1851, date: April 5, 2019) and was performed in accordance with the Declaration of Helsinki. All participants were informed of the benefits and risks of participating in the study prior to obtaining written informed consent. All participants signed an institutionally approved informed consent form.

The minimum sample size was initially calculated as 25 participants, with an α level of 0.05 (two sided), power of 0.90, effect size (f^2^) of 0.5 (large) [8], and 2–11 independent variables included in the multiple regression analysis [9]. Twenty-eight male athletes from the sports club in Juntendo University Faculty of Sport and Health Science participated in this study from July to August 2019. (a) Male college students, (b) those who joined the club to improve competitiveness, and (c) those without current severe clinical condition were included in the study. The athletes participated in track and field (throw and decathlon), handball, and basketball competitions. The participants were recruited though club announcements. The screening procedure included assessment of exercise history and self-reported medical history. In addition, the height, weight, and body composition were assed using dual-energy X-ray absorptiometry [DXA]), EI using BMR, and blood components using blood tests after a certain period of fasting. A 2-week dietary survey was conducted. Two participants whose blood samples showed abnormal values were excluded. Finally, the data of 26 participants were analyzed.

### Anthropometrics and DXA

Total body mass was measured to the nearest 0.1 kg on a physician’s scale (HBF-212, TANITA Inc., Tokyo, Japan), while height was measured to the nearest 0.1 cm using a stadiometer (YG-200, YAGAMI Inc., Nagoya, Japan). The body composition (body fat percentage and fat-free mass [FFM]) was measured by a trained radiologist using a Hologic QDR 4500 DXA scanner (Hologic, Inc., Bedford, MA). The intra- and inter-instrument reliabilities of DXA method have already been reported in previous studies [10, 11].

The equipment was calibrated daily according to the manufacturer’s instructions. All scans were analyzed using the Hologic QDR version 12.1 software (Hologic, Inc.). Based on the results of the DXA analyses, the head area was excluded, and the FFM and body fat mass were determined. To determine the technician’s error while using the software to estimate the body composition, the technician analyzed ten whole-body scans twice using the same method. Based on the results of the measurement, the technical errors (absolute and relative errors) were following: FFM (0.067 kg and 0.11%) and fat mass (0.070 kg and 0.76%).

### Three-day dietary record

Trained registered dietitians provided participants with written and verbal instructions on how to conduct the 3-day dietary record (DR) [12]. Dietary intake analysis was performed by a certified sports dietitian, regardless of the participants’ sports club, and the results were not shared with the team leaders. The participants were requested to report their dietary intake in an honest manner.

As part of the DR, the participants were requested to record the mealtime, location, and all foods and drinks consumed (except for water) in 3 consecutive days. To maximize the feasibility, intake was recorded for 3 days, 2 days with training, and 1 day without training, but not on days with special events (e.g., birthdays or championship match days). The DR form comprised mealtime, meal location, name of the dish, ingredients in the dish, and the total amount of food consumed. The participants were requested to record the food and drinks they consumed from the time they woke up until the time they went to bed, including supplements and beverages.

In addition, detailed information on dressings (presence or absence of oil, etc.), dairy products (low-fat milk, etc.), and intake amounts was also recorded. The participants were requested to record as much information as possible, including the consumed portion size and details of any leftovers, using household measurements (e.g., cups, pieces, tablespoons, and weight). Concurrently, the participants took photographs of all food and drinks alongside scale cards (length: 9 cm; width: 5.5 cm, with 1-cm graduations) using their smartphone cameras. For purchased food items, additional photographs that included the product name and food label were requested. The photographs were immediately sent to the specified E-mail addresses.

Based on the DRs and meal photographs, a registered dietitian (certified sports dietitian) analyzed the energy and nutrient intake using a nutritional analysis software (Calorie Make, version 1.0.10 and Nutrition Navigation, version 5.3.0; Toyo System Science Co., Ltd., Kanagawa, Japan).

### Determination of basal metabolic rate

The FFM measured by DXA was considered as lean body mass (LBM). The BMR was estimated based on the calculated LBM, using the following Japan Institute of Sports Sciences (JISS) formula: 28.5 kcal/kg LBM [13]. The EI/BMR ratio was set as the outcome variable.

### Blood samples

Fasting blood samples were collected from the antecubital vein without stasis. The parameters analyzed included a total of 36 items related to the following: serum protein, amino acid and nitrogen-compound, iron metabolism, serum enzyme, glucose metabolism, serum lipid, blood cell, pituitary hormone, thyroid hormone, adrenal cortex hormone, gonadal hormone, and other bioactivities. All assays were performed at a commercially available laboratory (SRL Inc., Tokyo, Japan).

### Statistical analysis

SPSS Statistics version 26.0 (IBM Corp., Armonk, NY) was used for all statistical analyses. Descriptive statistics were calculated for each variable and were indicated as the mean (standard deviation [SD]) and the median (first and third quartiles, Q1 and Q3) values. The normality of the data distribution was verified using the Shapiro–Wilk test. Non-normally distributed variables were then log-transformed and used in subsequent analyses.

From the 36 selected blood biomarkers, we confirmed and excluded the pairwise correlation when the Pearson’s correlation coefficient between two independent variables was > 0.6. As the result, 18 independent variables were preselected, and dietary EI/BMR variables were included in the multiple regression (the stepwise) analysis as predictor variables to obtain the best model for predicting EI/BMR based on the selected biomarkers.

Before multiple regression, multicollinearity was assessed for each independent variable. Multicollinearity was evaluated using the variance inflation factor (VIF), which was defined as the inverse of tolerance. A VIF of > 5.0 indicates multicollinearity between two variables in a regression model [14]. The degree of agreement of the estimation formula and dietary assessment results was confirmed by carrying out a Bland–Altman analysis [15]. A *p*-value of < 0.05 was considered significant. The data for regression analysis conformed to the assumptions of homoscedasticity, independence, normality, and linearity.

## Results

### Physical characteristics and nutrient intake

The participants’ physical characteristics and nutrient intakes are presented in Table 1.

**Table 1:** Physical characteristics and nutrient intake of the participants (*n* = 26)

Characteristic	Mean (SD)	Median (Q1, Q3)
Age (years)	19.6 (1.4)	19 (18, 21)
Height (cm)	175.6 (5.7)	176.4 (171.2, 180.0)
Weight (kg)	74.6 (10.6)	70.5 (67.3, 78.2)
Body mass index (kg/m^2^)	24.2 (3.4)	24.1 (21.4, 26.2)
Percentage body fat (%)	12.0 (2.9)	11.1 (9.6, 14.3)
Fat mass (kg)	9.21 (3.32)	8.18 (6.82, 10.42)
Fat-free mass (kg)	63.1 (7.8)	61.1 (57.7, 66.6)
Basal metabolic rate (kcal/d)	1,799 (223)	1,741 (1,644, 1,898)
Total energy intake (kcal/d)	3,103 (455)	3,121 (2,755, 3,446)
EI/BMR	1.74 (0.31)	1.68 (1.52, 1.91)
Protein intake (g/d)	103.2 (21.2)	101.3 (86.8, 119.3)
Fat intake (g/d)	90.2 (17.9)	89.6 (78.1, 99.8)
Carbohydrate intake (g/d)	447.2 (104.8)	442.1 (359.7, 532.3)

Values are presented as the mean (standard deviation [SD]) and median (first and third quartile, Q1, Q3)

*EI/BMR* energy intake/basal metabolic rate

### Multiple regression analyses of the EI/BMR estimation formula

Table 2 shows the 36 biomarkers and their characteristics. Results of the Shapiro–Wilk test and Pearson’s correlation coefficient are shown. The correlation of aspartate transaminase, alanine transaminase, insulin, hemoglobin A1C (HbA1c), low-density lipoprotein cholesterol (LDL-C), triglycerides (TGs), white blood cell (WBC) count, and leptin with EI/BMR was analyzed based on the logarithmically transformed values as they were regarded as having non-normal distributions. Total cholesterol, LDL-C, WBC, and free triiodothyronine (free T3) had a significant correlation with EI/BMR (Table 2). No extreme outliers were observed.

**Table 2:** Blood biomarker characteristics of the participants (*n* = 26)

Blood biomarker	Mean (SD)	Median (Q1, Q3)	Shapiro–Wilk test	Correlation^a^	
			*p*	r	*p*
**Serum protein**					
Total protein (g/dL)	7.1 (0.36)	7.1 (6.8, 7.2)	0.539	-0.077	0.710
Albumin (g/dL)	4.47 (0.21)	4.4 (4.3, 4.6)	0.151	-0.234	0.250
Pre-albumin (mg/dL)	27.0 (4.82)	27.1 (23.6, 29.9)	0.871	-0.171	0.404
**Amino acid and nitrogen-compound**					
Uric acid (mg/dL)	6.2 (1.1)	6.2 (5.4, 7.1)	0.751	-0.025	0.902
eGFRcreat (mL/min)	95.3 (10.9)	94.6 (88.5, 103.4)	0.804	0.030	0.884
Creatinine (mg/dL)	0.89 (0.09)	0.90 (0.83, 0.94)	0.908	-0.022	0.917
Urea nitrogen (mg/dL)	13.1 (2.6)	12.6 (11.4, 14.6)	0.498	-0.119	0.564
**Iron metabolism**					
Transferrin (mg/dL)	235 (29)	235 (217, 261)	0.676	0.074	0.718
Ferritin (ng/dL)	87.9 (42.3)	92.3 (45.6, 123.3)	0.510	-0.293	0.147
Total iron-binding capacity (μg /dL)	314 (32)	317 (286, 337)	0.438	0.128	0.532
Unsaturated iron-binding capacity (μg /dL)	191 (60)	198 (149, 228)	0.436	0.038	0.853
Iron (μg/dL)	124 (47)	114 (94, 154)	0.070	0.039	0.850
**Serum enzyme**					
Aspartate transaminase (U/L)^b^	23 (6)	23 (19, 25)	0.003	0.353	0.077
Alanine transaminase (U/L)^b^	18 (9)	17 (13, 22)	0.012	0.136	0.507
γ-glutamyl transpeptidase (U/L)	18 (4)	18 (15, 20)	0.238	0.300	0.137
**Glucose metabolism**					
Glucose (mg/dL)	88 (7)	88 (84, 93)	0.286	0.099	0.631
Insulin (μIU/mL)^b^	6.76 (2.86)	5.93 (5.24, 8.03)	0.008	-0.293	0.147
HbA1c (%)^b^	5.0 (0.2)	5.0 (4.9, 5.2)	0.016	-0.088	0.668
**Serum lipid**					
Total cholesterol (mg/dL)	161 (28)	157 (136, 181)	0.182	-0.416	0.034
High-density lipoprotein cholesterol (mg/dL)	57 (11)	54 (49, 67)	0.420	0.074	0.720
Low-density lipoprotein cholesterol (mg/dL)^b^	96 (25)	89 (76, 111)	0.025	-0.469	0.016
Triglycerides (mg/dL)^b^	76 (37)	63 (49, 91)	0.007	-0.366	0.066
**Blood cell**					
White blood cell count (/μL)^b^	5885 (1450)	5450 (4850, 6425)	0.041	-0.482	0.013
Red blood cell count (× 10^4^ /μL)	507 (24)	507 (489, 520)	0.684	-0.218	0.285
Hemoglobin (g/dL)	15.3 (0.6)	15.3 (15.0, 15.6)	0.210	-0.132	0.520
Hematocrit (%)	45.1 (1.7)	45.5 (44.1, 45.9)	0.074	-0.186	0.362
Mean corpuscular volume (fL)	88.9 (2.51)	89.1 (87.5, 90.6)	0.485	-0.112	0.587
Mean corpuscular hemoglobin (pg)	30.1 (1.2)	30.0 (29.4, 31.3)	0.496	-0.021	0.917
Mean corpuscular hemoglobin concentration (%)	34.0 (0.9)	34.0 (33.2, 34.6)	0.431	0.088	0.668
Platelet count (× 10^4^ /μL)	24.7 (3.4)	24.8 (22.5, 27.2)	0.857	0.051	0.803
**Pituitary hormone**					
Insulin-like growth factor 1 (ng/mL)	217 (54)	207 (180, 249)	0.163	-0.327	0.103
**Thyroid hormone**					
Free triiodothyronine (pg/mL)	3.81 (0.32)	3.75 (3.58, 4.01)	0.160	0.602	0.001
**Adrenal cortex hormone**					
Cortisol (μg/dL)	11.43 (2.60)	11.70 (8.83, 13.48)	0.200	0.168	0.411
**Gonadal hormone**					
Estradiol (pg/mL)	33.2 (11.0)	32.9 (26.2, 39.5)	0.779	0.020	0.921
Testosterone (ng/mL)	6.70 (1.95)	6.53 (5.33, 8.47)	0.901	0.185	0.366
**Other bioactivity**					
Leptin (ng/mL)^b^	4.6 (1.7)	4.4 (3.3, 5.2)	0.007	-0.182	0.374

Values are presented as the mean (standard deviation [SD]) and the median (first and third quartile, Q1, Q3)

^a^ Dependent variables: dietary energy intake/basal metabolic rate

^b^ The correlation coefficient is calculated using the logarithmically transformed values

eGFRcreat: estimated glomerular filtration rate, HbA1c: hemoglobin A1c

The stepwise methods included the following measurements: levels of albumin, uric acid, estimated glomerular filtration rate, urea nitrogen, ferritin, aspartate aminotransferase (logarithmic conversion), insulin (logarithmic conversion), HbA1c (logarithmic conversion), high-density lipoprotein cholesterol, LDL-C (logarithmic conversion), TGs (logarithmic conversion), WBCs (logarithmic conversion), red blood cells, somatomedin C, free T_3_, cortisol, testosterone, and leptin (logarithmic conversion). The stepwise regression analysis was performed to develop a formula for estimating the EI/BMR based on the results of blood tests.

Table 3 presents a summary of the extracted models 1–3. Model 1 consisted of free T3. Model 2 consisted of free T3 and WBC. Model 3 consisted of free T3, WBC, and TGs. All multiple regression equations were significant (all *p* < 0.001). In the final model (model 3), the three significant biomarkers were retained, with standard regression coefficients of magnitude A, B, and C, in that order, and with 60.4% of the total variance accounting for the dietary EI/BMR. The VIF of the selected variables was 1.00–1.01, and no multicollinearity was observed. Therefore, a new formula was developed:

**Table 3:** Multiple regression analysis model for estimating the energy intake/basal metabolic rate

	Model 1				Model 2				Model 3			
	β,	SE (β)	Standardized β		β,	SE (β)	Standardized β		β,	SE (β)	Standardized β	
Constant	− 0.489	0.607	-		4.510	1.720	-		5.524	1.590	-	
Free T3 (pg/mL)	0.586	0.159	0.602	***	0.548	0.137	0.562	***	0.526	0.123	0.540	***
White blood cell count (/μL)^a^	-	-	-		− 1.292	0.423	− 0.431	**	− 1.291	0.379	− 0.430	**
Triglycerides (mg/dL)^a^	-	-	-		-	-	-		− 0.507	0.197	− 0.326	*
F	13.614	***			13.821	***			13.698	***		
Adjusted R^2^	0.335	**			0.506	***			0.604	***		

*β* partial regression coefficient, *SE(β)* standard error of the partial regression coefficient, standardized, *β* standardized partial regression coefficient, *T3* triiodothyronine

Dependent variable: energy intake/ basal metabolic rate

Only variables identified as significant independent variables by stepwise method are used

^*^p < 0.05, **p < 0.01, ***p < 0.001

^a^Log-transformed parameters

$$\mathrm{Estimated\, EI}/\mathrm{BMR}=0.526 \times \mathrm{ free \,}{\mathrm{T}}_{3} (\mathrm{pg}/\mathrm{mL})-1.291\times {\mathrm{log}}_{10}\mathrm{ WBC \,}(/\mathrm{\mu L})-0.507\times {\mathrm{log}}_{10}\mathrm{ TGs\, }(\mathrm{mg}/\mathrm{dL})+5.524$$

**Fig. 1:**
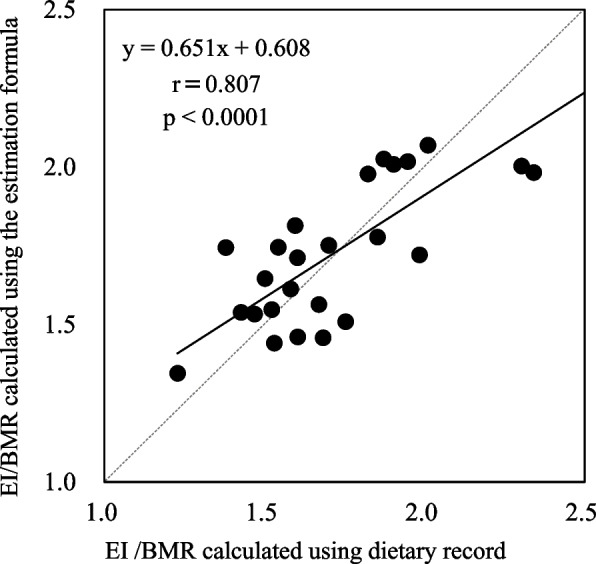
Relationship between energy intake and basal metabolic rate calculated using the estimation formula and dietary record. EI/BMR: energy intake/basal metabolic rate. The solid line represents the regression line. The semi-continuous line represents the identity line

**Fig. 2:**
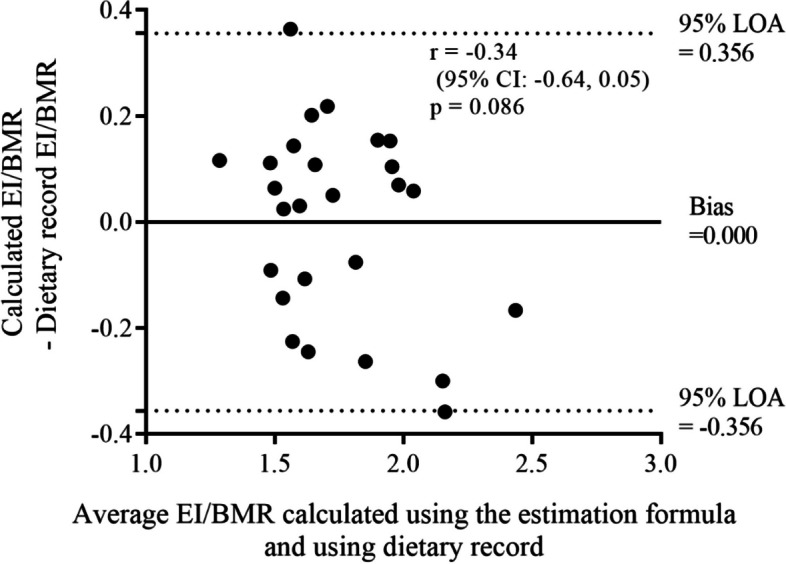
Bland–Altman plot of energy intake and basal metabolic rate calculated using the estimation formula and dietary record. EI/BMR: energy intake/basal metabolic rate. 95% CI: confidence intervals for correlation coefficients. LOA: limits of agreement

The mean (SD) and median (first and third quartile, Q1 and Q3) values of the estimated EI/BMR were 1.74 (0.24) and 1.73 (1.53–1.98), respectively. Figure 1 shows the EI/BMR calculated using the estimated equations included in the final model (model 3) (Fig. 1). Bland–Altman analysis confirmed the absence of systematic errors in the estimated values calculated using the estimation formula and dietary assessment results (*p* = 0.086) (Fig. 2).

## Discussion

To the best of our knowledge, this is the first study to identify the blood biomarkers for estimating EI/BMR in male Japanese athletes. The main findings were as follows: (1) free T_3_, WBC, and TGs were independent predictors of EI/BMR, and (2) these three biomarkers were used, in that order, to develop an EI/BMR estimation formula based on the results of the multiple regression analysis. The predictive ability of the developed formula was 60.4%, in relation to estimating the EI/BMR using a DR. Although some blood biomarkers are known to pathologically and accurately represent low and high EI [16–18], this is the first study to estimate the EI with high accuracy by combining multiple blood biomarkers.

A previous relevant study observed changes in blood biomarkers at low EI in soldiers who underwent an 8-week United States Army Ranger training, which consisted of four repeated cycles of restricted EI and refeeding. In this study, low concentrations of free T_3_ were found to be reliable biomarkers of energy deficiency in healthy young men [16]. Additionally, well-controlled laboratory experiments have demonstrated that energy deficiency is associated with the suppression of the expression of key metabolic hormones such as T_3_ and free T_3_ [19]. Therefore, free T3 was selected as a candidate biomarker in this study because low EI was associated with lower free T3 concentrations in male athletes, as reported by these previous studies.

Previous research suggests that free T3 alone is likely to be associated with EI, but our study revealed that combining multiple biomarkers could improve the accuracy of EI measurement. In our study, the predictive ability of the developed formula that only included free T_3_ concentration (model 1) was 33.5%, while that of the formula that included free T_3_ concentration, WBC, and TG levels (model 3) was 60.4%, in relation to the estimation of EI/BMR using a DR. However, to the best of our knowledge, no correlation between WBC, TG and energy deficiency has been reported in healthy individuals. Moreover, the levels of blood biomarkers used for these predictions were within normal. By adding a few items, a significantly higher estimation power was obtained, which clearly led to a more accurate EI/BMR estimation.

Estimation of dietary intake on a regular basis using these blood biomarkers may allow a wider range of individuals to know their EI, even in teams without sports dietitians. Additionally, self-reported dietary assessments involve the inability to fully and accurately recall intake, limitations inherent in doing conversions in the food composition databases and are issues related to underestimation and overestimation [2, 20, 21]. The use of these biomarkers may rectify the EI calculated by such dietary survey.

Moreover, dietary assessment using DRs places a heavy burden on athletes and has reduced reliability over time. Conversely, biomarkers are stable, and its reliability can be maintained [22]; further, the captured data do not depend on the knowledge of athletes or investigators. The objective estimation of nutritional status is often recommended to overcome DR errors and better capture the intra-individual variability in EI [23, 24]; therefore, EI determination using blood biomarkers may be a feasible option.

This study has several limitations. First, the accuracy of the diet survey could not be evaluated using DLW [24]. The DLW method is a biomarker of energy expenditure that can be used to validate the self-reported EI [24]. Second, a self-reported dietary assessment method was conducted. The self-reported data on EI cannot be used to measure the true EI [25, 26]. However, we employed all possible measures to prevent under- and overreporting. Specifically, real-time photograph submission via the web was requested to reduce the burden on participants, and the days with special occasions (match day) among the DR days were excluded. To avoid intentional errors in reporting as much as possible, a sports dietitian independent of the club collected and analyzed the DR. Although it is still uncertain that the equation in this study can estimate the true EI, it is able to capture the same level of EI determined by a trained sports dietitian who conducted a careful nutritional survey.

Third, the participants only included collegiate athletes of track and field (throw/decathlon) events, handball, and basketball. The metabolic properties vary among different sports athletes, and we did not verify whether the formula was suitable for all athletes. Fourth, this study included male athletes alone. It is possible for the blood biomarkers for estimating EI/BMR to be impacted by the menstrual cycle in female athletes. To avoid this effect, male athletes were used in this study.

Finally, this cross-sectional study was conducted in a limited sample as a pilot study. In future studies, it will be necessary to track the changes associated with intra-individual variability in these blood biomarkers to more reliably estimate the EI. In addition, the accuracy of the formula in other populations should also be analyzed. The value obtained by the estimation formula using the blood biomarkers does not represent the absolute dietary intake. Therefore, the formula should only be used for estimating the EI/BMR and determining relative intake within the population. The estimation formula is useful to compensate for the shortcomings of current methods, especially DR reporting errors. Future investigations should focus on the components of EI/BMR and the actual energy availability.

## Conclusion

The combination of free T3 level, white blood cell count, and triglyceride level can be used for estimating the EI/BMR of male athletes. This method helps in overcoming the shortcomings of dietary surveys.

## Data Availability

The datasets used and/or analysed during the current study are available from the corresponding author on reasonable request.

## Abbreviations

EI: Energy intake
BMR: Basal metabolic rate
FFM: Fat-free mass
LBM: Lean body mass
JISS: Japan Institute of Sports Sciences
VIF: Variance inflation factor
TGs: Triglycerides
WBC: White blood cell
DR: Dietary record
